# AQP3 and AQP5 Modulation in Response to Prolonged Oxidative Stress in Breast Cancer Cell Lines

**DOI:** 10.3390/antiox13060626

**Published:** 2024-05-21

**Authors:** Monika Mlinarić, Ivan Lučić, Marko Tomljanović, Ivana Tartaro Bujak, Lidija Milković, Ana Čipak Gašparović

**Affiliations:** 1 Laboratory for Membrane Transport and Signaling, Division of Molecular Medicine, Ruđer Bošković Institute, HR10000 Zagreb, Croatia; monika.mlinaric@irb.hr (M.M.); ivan.lucic@irb.hr (I.L.); marko.tomljanovic@irb.hr (M.T.); lidija.milkovic@irb.hr (L.M.); 2 Radiation Chemistry and Dosimetry Laboratory, Division of Materials Chemistry, Ruđer Bošković Institute, HR10000 Zagreb, Croatia; ivana.tartaro.bujak@irb.hr

**Keywords:** AQP3, AQP5, oxidative stress, breast cancer

## Abstract

Aquaporins are membrane pores regulating the transport of water, glycerol, and other small molecules across membranes. Among 13 human aquaporins, six have been shown to transport H_2_O_2_ and are therefore called peroxiporins. Peroxiporins are implicated in cancer development and progression, partly due to their involvement in H_2_O_2_ transport. Oxidative stress is linked to breast cancer development but is also a mechanism of action for conventional chemotherapy. The aim of this study is to investigate the effects of prolonged oxidative stress on Aquaporin 3 (AQP3), Aquaporin 5 (AQP5), and signaling pathways in breast cancer cell lines of different malignancies alongside a non-tumorigenic breast cell line. The prolonged oxidative stress caused responses in viability only in the cancer cell lines, while it affected cell migration in the MCF7 cell line. Changes in the localization of NRF2, a transcription factor involved in oxidative stress response, were observed only in the cancer cell lines, and no effects were recorded on its downstream target proteins. Moreover, the prolonged oxidative stress caused changes in AQP3 and AQP5 expression only in the cancer cell lines, in contrast to their non-malignant counterparts. These results suggest peroxiporins are potential therapeutic targets in cancer treatment. However, further research is needed to elucidate their role in the modulation of therapy response, highlighting the importance of research on this topic.

## 1. Introduction

Aquaporins are a family of integral membrane proteins that play a fundamental role in the regulation of water transport across the cell membrane in response to osmotic gradients and are responsible for maintaining cell water homeostasis [1,2]. Thirteen different human aquaporin isoforms have been identified and are grouped based on their primary sequence and permeability into three groups: water-selective orthodox aquaporins (AQP0, AQP1, AQP2, AQP4, AQP5, AQP6, and AQP8), aquaglyceroporins (AQP3, AQP7, AQP9, and AQP10) which are selective for water and other small uncharged molecules such as glycerol and urea [3], and unorthodox aquaporins (AQP11 and AQP12) with a deviation in the NPA motif specific to the aquaporin family and exclusively located inside cells [4]. Another important substrate of aquaporins is hydrogen peroxide (H_2_O_2_) [5], a reactive oxygen species (ROS) and an important second messenger involved in redox signaling in the cell [6]. Due to their structure, it is hypothesized that all aquaporins are H_2_O_2_ channels [7], but only some of them have been proven as such (AQP1, AQP3, AQP5, AQP8, AQP9, and AQP11) [8,9], and this group of aquaporins is named peroxiporins [10]. In addition to the previously mentioned roles, recent research has shown that aquaporins are multifunctional proteins that contribute to various cellular processes, including cell migration, proliferation, and angiogenesis [11]. Their expression and activity are tissue-specific and finely tuned for physiological demands; for example, in the kidneys, they participate in urine concentration, in the eye, they maintain the transparency of the lens, they maintain water homeostasis in the brain, control the amount of glycerol in fat metabolism, etc. [3,4,11]. The complicated regulation of aquaporins’ expression and activity highlights their importance in various physiological processes and contributes to the functionality of tissues, so it is not surprising that aquaporin dysregulation is often observed in different pathological conditions, including cancer.

The International Agency for Research on Cancer (IARC) estimated there were 19.3 million cancer cases and 10 million cancer deaths in 2020, with breast cancer being the most commonly diagnosed cancer among women worldwide [12]. Even though there have been improvements in chemo-, radio-, and targeted therapies, relapse-free survival is still a challenge. Breast cancer continues to be a subject of research, and new biomarkers and therapeutic targets are constantly being discovered, with aquaporins being one of them. Numerous studies have already shown a correlation between aquaporin expression and tumor type, grade, and prognosis, as well as the development of drug resistance, but it is still not clear if aquaporins could be considered therapeutic targets or biomarkers [13,14]. The upregulation of different aquaporins, specifically peroxiporins, has been shown in numerous cancers, with AQP1, AQP3, and AQP5 being the most common and associated with cancer progression and changes in chemotherapy sensitivity [15]. For breast cancer, AQP3 and AQP5 are the most frequently mentioned as potential therapeutic targets and/or biomarkers [16,17,18,19].

Cancer is intricately associated with oxidative stress, which is one of the driving forces in its development and progression, while it is also used as a therapeutic mechanism to counteract it. Breast cancer has a high oxidative stress burden due to estrogen-mediated and therapy-induced ROS production. These increased levels of ROS are recognized as one of the key factors that support several hallmarks of cancer [20], and to protect against oxidative damage and apoptosis, cancer cells upregulate many antioxidants and cytoprotective mechanisms. As the first line of defense against oxidative stress, the transcription factor NRF2 (nuclear factor erythroid 2-related factor 2), a master regulator of the expression of many cytoprotective genes, is constitutively active in breast cancer and associated with cancer progression and resistance to therapy [9,21]. Furthermore, the most aggressive subtypes of triple-negative breast cancer overexpress SLC7A11, a direct target of NRF2 [22]. This overexpression increases cystine uptake, which in turn inactivates Keap1 via the cysteinylation of residues 226 and 613, and activates NRF2, enhancing resistance to oxidative stress [22]. This resistance provides protection while enabling cancer cells to abuse high levels of ROS for their proliferation and progression. Among ROS, hydrogen peroxide is produced under physiological and also pathological conditions. It is an important part of various cellular signaling pathways [23]. NADPH oxidases (NOX) produce H_2_O_2_ to support signaling pathways [24,25], and this process is mediated by AQP3 [26]. In addition to autocrine secretion, cancer cells are exposed to oxidative stress caused by inflammation [27] or cancer therapy [28]. Oxidative stress in the tumor microenvironment may play a crucial role in cancer aggressiveness. Namely, the chronic exposure of MCF7, a breast cancer cell line, to H_2_O_2_ increases its tumorigenic potential [29], thereby underscoring the role of H_2_O_2_ in cancer. Since aquaporins play a role in H_2_O_2_ transport and are upregulated in cancer, and cancer has an increased cellular ROS level, could aquaporins be one of the players maintaining the redox balance in cells and affecting cancer progression?

To address this question, in this study, we simulated elevated ROS levels with H_2_O_2_ treatments for two weeks in three different breast cancer cell lines: hormone-positive MCF7, HER2-positive SkBr3, and triple-negative SUM159PT, as well as the MCF10A non-tumorigenic breast epithelial cell line, and observed their adaptation to induced, prolonged oxidative stress. Understanding the mechanisms by which cancer cells adapt to elevated ROS conditions and how aquaporins affect this response is essential for understanding their role in cells, as well as discovering why they are often upregulated in cancer and whether they could be therapeutic targets.

## 2. Materials and Methods

### 2.1. Cell Lines

Three human breast cancer cell lines (MCF7, SkBr3, and SUM159PT) and one human non-tumorigenic breast epithelial cell line (MCF10A), purchased from EACC (Porton Down, UK) or Elabscience (Wienna, Austria), were used in this study. MCF7 is an estrogen (ER) and progesterone receptor (PR)-positive cell line, SkBr3 is an HER2-positive cell line, and SUM159PT is a triple-negative (ER-, PR-, and HER2-negative) cell line. The cancer cell lines were cultivated in DMEM (Sigma Aldrich, St. Louis, MO, USA) supplemented with 10% fetal calf serum (FCS, Sigma Aldrich), while the non-tumorigenic cell line was cultured in DMEM:F12 1:1 (Sigma Aldrich) containing 10% FCS, 10 µg/mL insulin, 20 ng/mL epidermal growth factor (EGF, PeproTech, London, UK), and 100 ng/mL cholera toxin (Sigma Aldrich). Cells were grown in a humidified atmosphere with 5% CO_2_ at 37 °C. Upon reaching semiconfluency, cells were trypsinized, counted, and seeded for treatments.

### 2.2. Cell Culture Treatments

To assess the influence of prolonged exposure to oxidative stress, 3 × 10^5^ cells were seeded in a T25 flask (TPP, Trasadingen, Switzerland) and were treated with 10 or 20 µM hydrogen peroxide (H_2_O_2_) for 14 days, with media changes every two days. Control cells were grown in parallel, undergoing the same seeding, culture, and media changes, but without adding H_2_O_2_. During these 14 days, cells were trypsinized on the day between the treatments when they achieved a confluence of about 80%. Analyses were conducted following their exposure to H_2_O_2_. For protein analyses, cells were harvested in RIPA buffer (ThermoFisher Scientific, Waltham, MA, USA); for mRNA analyses, cells were harvested in TRIzol (ThermoFischer Scientific); and for lipid and lipid hydroperoxide (LOOH) analyses, cells were trypsinized, centrifuged, and stored as dry pellet till analyses were performed.

### 2.3. Viability and Proliferation Assays

For cell viability and proliferation assays, 1 × 10^4^ cells per well were seeded in a 96-well plate. After allowing the cells to attach for 24 h, they were exposed to H_2_O_2_ at concentrations ranging from 0 to 100 µM. Concentrations were selected ranging from physiologic to pathologic values to test if low doses of H_2_O_2_ triggered adaptive response. Following an additional 24 h of incubation, cell viability was assessed using an EZ4U MTT assay (Biomedica, Vienna, Austria), while cell proliferation was evaluated with a BrdU assay (Roche, Basel, Switzerland). Both assays were carried out following the manufacturers’ instructions. Briefly, the MTT assay evaluates cell viability by measuring metabolic activity. The oxidation of the MTT dye leads to a transformation from a colorless to a water-soluble yellow product, which is quantified using a plate reader (EZ Read 2000, Biochrom, Cambridge, UK) at 450 nm, with a reference wavelength of 620 nm. The BrdU assay measures cell proliferation by incorporating 5-bromo-2′-deoxyuridine (BrdU) into newly synthesized DNA. Detection is achieved with an anti-BrdU-POD solution, and subsequent color development is stopped with a stop solution. The resulting color change is then measured using a plate reader at 450 nm.

### 2.4. Total Lipid Extraction and GC Analysis

The extraction of lipids from dry cell pellets was conducted using the modified Folch method [30]. For this purpose, 5 mL of chloroform was introduced into the samples and blended thoroughly. Following this, an aqueous solution of MgCl_2_ (1.5 mL; 0.034%, *w*/*v*) was added to the samples, which were then subjected to vortexing and centrifugation. The upper aqueous phase was carefully discarded, and a 2 M solution of KCl in methanol (2.5 mL; 4:1, *v*/*v*) was introduced. After vortexing and centrifugation, the aqueous phase was discarded, and a chloroform/methanol solution (2.5 mL; 2:1, *v*/*v*) was added. The resulting hydrophobic phase was meticulously collected and transferred to a fresh tube, with subsequent removal of the solvent through evaporation under a nitrogen stream. To convert the lipids into fatty acid methyl esters, a 0.5 M solution of KOH in methanol was applied to the lipid extracts for 10 min at room temperature. The resulting fatty acid methyl esters were then extracted using n-hexane and subjected to analyses via gas chromatography (GC). The GC analyses of total fatty acids were performed utilizing a Varian 450-GC equipped with a flame ionization detector. A Stabilwax column (crossbond carbowax polyethylene glycol, 60 m × 0.25 mm) was employed as the stationary phase, with helium serving as the carrier gas. The temperature was ramped from 150 °C to 250 °C at a rate of 5 °C/min. Identification of the methyl esters was achieved by comparing their retention times with those of commercially available standard mixtures (marine oil FAME mix, Restek Corporation, Bellefonte, PA, USA). Quantification of the FAME percentage (relative amount of each fatty acid) was accomplished by integrating the area under the peak and dividing the results by the total area for all fatty acids.

### 2.5. Measurement of LOOH Concentration

The lipids were extracted following the method described previously (refer to Section 2.4). Subsequently, the organic layer containing the lipids was meticulously transferred to a glass tube, and the solvent was evaporated. After being weighed, the lipids were stored at −20 °C until the analysis of lipid hydroperoxides (LOOH) was conducted. The concentration of LOOH was determined utilizing a spectrophotometric ferric thiocyanate assay. The analyzed samples were diluted with a deaerated mixture of CH2Cl2/MeOH (2:1, *v*/*v*). The concentrations of LOOH were computed employing the molar absorptivity of the complex [FeNCS]^2+^ formed per mole of LOOH, which is 58,440 dm^3^ mol^−1^ cm^−1^, measured at 500 nm [31].

### 2.6. Cell Migration Assay

To evaluate the effect of prolonged exposure to H_2_O_2_ on cell migration, 3 × 10^4^ cells were seeded per well in a 96-well plate. After 24 h, cells were treated with 5 mg/mL mitomycin c for 2 h, after which the cells were scratched, and the medium was replaced with new one with and without H_2_O_2_. Images of the scratch were captured immediately post-scratching, after 24 h, and after 48 h. The surface of the wound was quantified using ImageJ software 1.53t.

### 2.7. RNA Isolation, cDNA Synthesis, and RT-qPCR

Following 14 days of H_2_O_2_ exposure, total RNA was isolated using TRIzol (Thermo Fisher Scientific, Waltham, MA, USA) following the instructions provided by the manufacturer. The purity and concentration of RNA were determined spectrophotometrically using NanoPhotometer^®^ N60 (Implen GmbH, München, Germany), and 1 µg of each RNA was transcribed to cDNA using a High-Capacity cDNA Reverse Transcription Kit (Thermo Fisher Scientific, following the instructions provided by the manufacturer) on an Eppendorf 5331 MasterCycler Gradient Thermal Cycler (Eppendorf, Hamburg, Germany). Two µL of cDNA template was used per reaction for quantitative reverse transcription PCR (RT-qPCR) that was performed on a CFX Opus 96 Real-Time PCR System (Bio-Rad Laboratories, Hercules, CA, USA) with TaqMan Universal PCR Master Mix (Thermo Fisher Scientific) and the following specific TaqMan predesigned gene expression assays: AQP3 (Hs01105469_g1), AQP5 (Hs00387048_m1), NFE2L2 (Hs00975961_g1), and ACTB (Hs01060665_g1) (Thermo Fisher Scientific). For quantitative determination of AQP1, AQP9, AQP11, and housekeepers B2M, PPIA, and HPRT-1 transcripts, SYBR Green chemistry was used, and primers were constructed, which are listed in Table 1. Each reaction was composed of 10 µL of SsoAdvanced Universal SYBR Green Supermix (Bio-Rad Laboratories, Hercules, CA, USA), 8 µL of deionized water, 0.5 µL mixed forward and reverse primers (5 µM each), and 1.5 µL of cDNA template. The protocol for amplification was as follows: initial incubation at 95 °C for 2 min, followed by 40 cycles of 95 °C for 15 s and 62 °C for 30 s. Relative quantification of gene expression was determined with the 2(-Delta Delta C(T)) method [32] and is shown as a fold change between treated and control conditions.

### 2.8. Protein Isolation and Western Blot Analyses

Following 14 days of H_2_O_2_ exposure, proteins were harvested in RIPA buffer containing protease and phosphatase inhibitors. In parallel, nuclear and cytoplasmic fractions were extracted using NE-PER Extraction Reagent (Thermo Fisher Scientific), according to the manufacturer’s instructions. Protein concentration was measured using the Bradford method [33], with bovine serum albumin (BSA) standards in either 20% RIPA buffer or in phosphate-buffered saline (PBS), depending on the isolation method. Subsequently, 20 µg of proteins was separated via SDS-PAGE on an 8% resolving gel and then transferred to a nitrocellulose membrane (Roti-NC 0.2 µm; Carl Roth, Karlsruhe, Germany). The membrane was stained with Ponceau S, scanned, and then blocked with 5% BSA. The following were used for overnight incubation at +4 °C: primary antibodies (anti-Nrf2 (D1Z9C), anti-HO-1 (E3F4S), anti-NQO1 (D6H3A), anti-GSK-3β (D5C5Z), anti-Keap1 (D6B12), anti-ABCB1 (E1Y7B), anti-ABCG2 (D5V2K), anti-PI3K (C73F8), anti-PTEN (D4.3), anti-p-Akt (D9E), anti-Akt (C67E7), anti-Ras (27H5), anti-phospho-mTOR (D9C2), anti-Raptor (24C12), anti-Rictor (53A2), anti-β-actin (D6A8), anti-LSD1 (2139) (1:1000, Cell Signaling Technology (CST), Danvers, MA, SAD); anti-AQP3 (sc-518001) and anti-AQP5 (sc-514022) (1:200, Santa Cruz Biotechnology, Dallas, TX, USA); and anti-AKR1B10 (ab96417) (1:10,000, Abcam, Cambridge, UK). After washing, the membrane was incubated with either anti-rabbit IgG, HRP-linked antibody (1:2000, 7074, CST), or anti-mouse IgG, HRP-linked antibody (1:4000, 96714, CST) for 1 h at room temperature. The signal was visualized with SuperSignal™ West Pico PLUS Chemiluminescent Substrate (Thermo Fisher Scientific) and the chemiluminescence was detected using the Alliance 4.7 Digital Imaging System (Uvitec, Cambridge, UK). Signal quantification was performed with the Nine Alliance software Q9 (Uvitec), and protein expression levels were normalized using housekeeping proteins (β-actin or LSD-1) and Ponceau S staining.

### 2.9. Statistical Analysis

Experiments were conducted in biological and technical triplicates, and results are expressed as mean ± SEM. Statistical analyses, including Student’s *t*-test, one-way ANOVA, and two-way ANOVA with Tukey or Dunnett’s test, were performed using Excel and GraphPad Prism software 8.0 (GraphPad Software, La Jolla, CA, USA). *p* values below 0.05 were considered statistically significant.

## 3. Results

To investigate the effect of prolonged exposure to oxidative stress on breast cancer cells, we used three different breast cancer cell lines, estrogen receptor (ER)- and progesterone receptor (PR)-positive MCF7, HER2-positive SkBr3, and triple-negative SUM159PT. We used a nontumorigenic breast epithelial cell line, MCF10A, as a control.

### 3.1. Effect of Prolonged Exposure to H_2_O_2_ on Cell Viability and Proliferation

**Figure 1:**
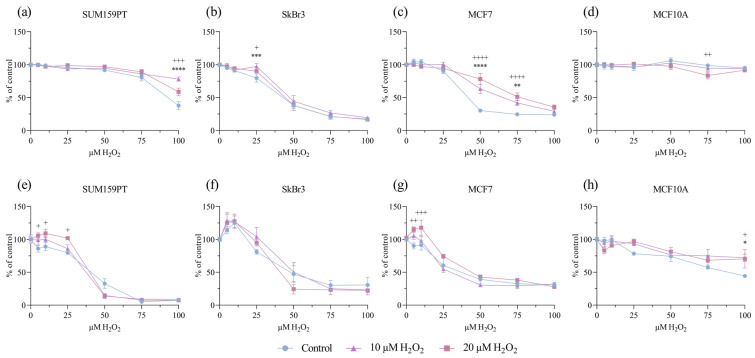
Effect of prolonged exposure to H_2_O_2_ on cell viability and proliferation. Cells were treated with 10 or 20 µM H_2_O_2_ for 14 days, after which they were treated with a range of H_2_O_2_ concentrations. After 24 h, cell viability and proliferation were assessed by MTT and BrdU assays. Cell viability is shown on panels (**a**) SUM159PT, (**b**) SkBr3, (**c**) MCF7, and (**d**) MCF10A. Cell proliferation is shown on panels (**e**) SUM159PT, (**f**) SkBr3, (**g**) MCF7, and (**h**) MCF10A. Experiments were performed biologically and technically in triplicate. Cell viability was calculated as the ratio between the treated cells and untreated control and is shown as a percentage of the control. The results are presented as mean ± SEM. The asterisk (*) indicates the *p* value for the 10 µM-treated cells compared to the control, and the plus (^+^) indicates the *p* value for the 20 µM H_2_O_2_-treated cells compared to the control, *^/+^ *p* ≤ 0.05, **^/++^ *p* ≤ 0.01, ***^/+++^ *p* ≤ 0.001, ****^/++++^ *p* ≤ 0.0001.

We examined the effect of prolonged exposure to low concentrations of H_2_O_2_ (10 and 20 µM) on cell viability and proliferation after an additional challenge with H_2_O_2_ in a range of concentrations. Cell viability was assessed using an MTT assay and proliferation using a BrdU incorporation assay. Prolonged treatments with both 10 and 20 µM H_2_O_2_ affected cell viability and/or proliferation. In the SUM159PT cell line, prolonged exposure to both concentrations of H_2_O_2_ caused an increase in the viability of the cells treated with 100 µM H_2_O_2_ compared to that of the control (*p* ≤ 0.0001, *p* ≤ 0.001) (Figure 1a), but only exposure to 20 µM H_2_O_2_ affected their proliferation, which increased in response to 5, 10, and 25 µM H_2_O_2_ (*p* = 0.0138, *p* = 0.0113, and *p* = 0.0118) (Figure 1e). The viability of SkBr3 cells was affected by the treatment with 25 µM H_2_O_2_, in prolonged exposure with 10 µM H_2_O_2_ (*p* ≤ 0.001), and with 20 µM H_2_O_2_ (*p* = 0.0485) (Figure 1b), but there were no changes in SkBr3 proliferation (Figure 1f). In the MCF7 cell line, exposure to 10 µM H_2_O_2_ caused an increase in viability in response to 50 and 75 µM H_2_O_2_ (*p* ≤ 0.001, *p* = 0.0073), with the same effect (both *p* ≤ 0.001) when exposed to 20 µM H_2_O_2_ (Figure 1c). MCF7 cell proliferation was increased only with the 20 µM H_2_O_2_ pretreatments, noticeably in response to 5 and 10 µM H_2_O_2_ (*p* = 0.0016, *p* ≤ 0.001) (Figure 1g). Interestingly, in the MCF10A cell line, a decrease in cell viability was observed in response to the 75 µM H_2_O_2_ treatment (*p* = 0.0071) (Figure 1d), but exposure to 10 and 20 µM H_2_O_2_ caused an increase in cell proliferation in response to the 100 µM H_2_O_2_ treatment (*p* = 0.018, *p* = 0.0266) (Figure 1h).

### 3.2. Effect of Prolonged Exposure to H_2_O_2_ on Cell Migration

**Figure 2:**
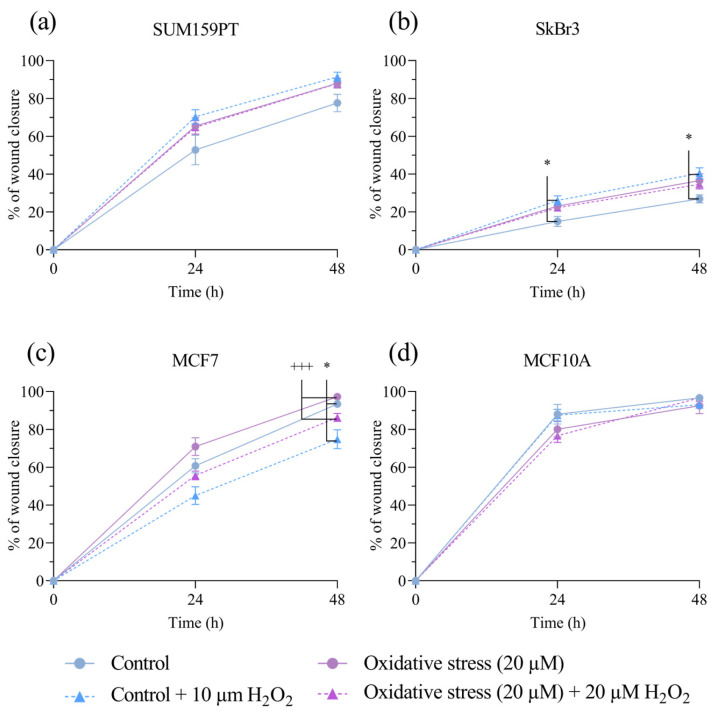
Effect of prolonged exposure to H_2_O_2_ on cell migration. (**a**) SUM159PT, (**b**) SkBr3, (**c**) MCF7, and (**d**) MCF10A cell lines were treated with 20 µM H_2_O_2_ for 14 days, after which they were scratched and treated with 20 µM H_2_O_2_. Cells were photographed after scratching as well as 24 and 48 h afterwards. Cell migration is calculated as the reduction in wound area over time, shown as a percentage of the starting wound area. Experiments were performed biologically and technically in triplicate. Results are presented as mean ± SEM. * *p* ≤ 0.05 compared to untreated control; ^+++^ *p* ≤ 0.001 compared to 20 µM H_2_O_2_-treated cells.

To investigate if exposure to prolonged oxidative stress affected cell migration, we performed a wound-healing assay, with the addition of mitomycin C to inhibit cell proliferation. Prolonged exposure to 20 µM H_2_O_2_ did not alter cell migration in either cell line. Wound healing was improved by acute treatment with 20 µM H_2_O_2_ in the MCF7 cell line after 48 h, both in the control cells (*p* = 0.0205) and in the cells with prolonged exposure (*p* ≤ 0.001) (Figure 2c). Additionally, an improvement in wound healing by acute H_2_O_2_ treatment was evident in the untreated SkBr3 cells at 24 (*p* = 0.0406) and 48 (*p* = 0.0110) hours (Figure 2b). SUM159PT and MCF10A showed faster rates of wound closure compared to those of the SkBr3 and MCF7 cell lines but were not affected by acute or prolonged exposure to H_2_O_2_.

### 3.3. Effect of Prolonged Exposure to H_2_O_2_ on Fatty Acid Content and LOOH Formation

**Figure 3:**
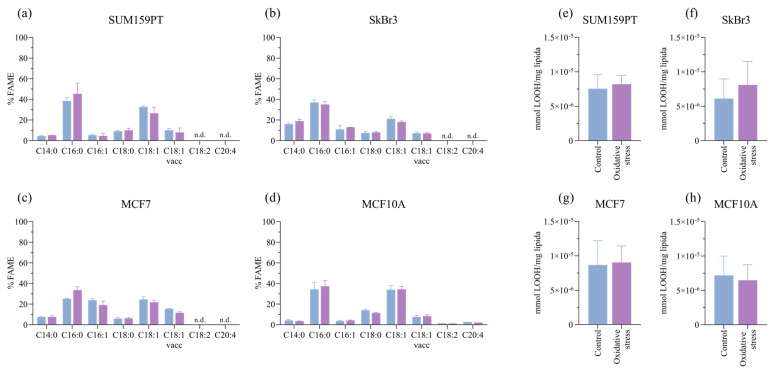
Effect of prolonged exposure to H_2_O_2_ on fatty acid content and lipid hydroperoxide (LOOH) formation. Cells were treated with 20 µM H_2_O_2_ for 14 days, after which cells were collected for analysis. The effect of H_2_O_2_ on lipid composition is shown in panels (**a**) SUM159PT, (**b**) SkBr3, (**c**) MCF7, and (**d**) MCF10A, and the effect on lipid hydroperoxide formation is shown in panels (**e**) SUM159PT, (**f**) SkBr3, (**g**) MCF7, and (**h**) MCF10A. Experiments were performed biologically and technically in triplicate. Results are presented as mean ± SEM.

Next, we analyzed the fatty acid content after prolonged exposure to oxidative stress. Oxidative stress causes the peroxidation of polyunsaturated fatty acids, with LOOH being intermediate products that are markers of intracellular oxidative stress levels. Although polyunsaturated fatty acids are substrates for peroxidation, the cells do not change significantly under prolonged stress (Figure 3a–d), and there is no effect on LOOH (Figure 3e–h).

### 3.4. Effect of Prolonged Exposure to H_2_O_2_ on Protein and Gene Expression

**Figure 4:**
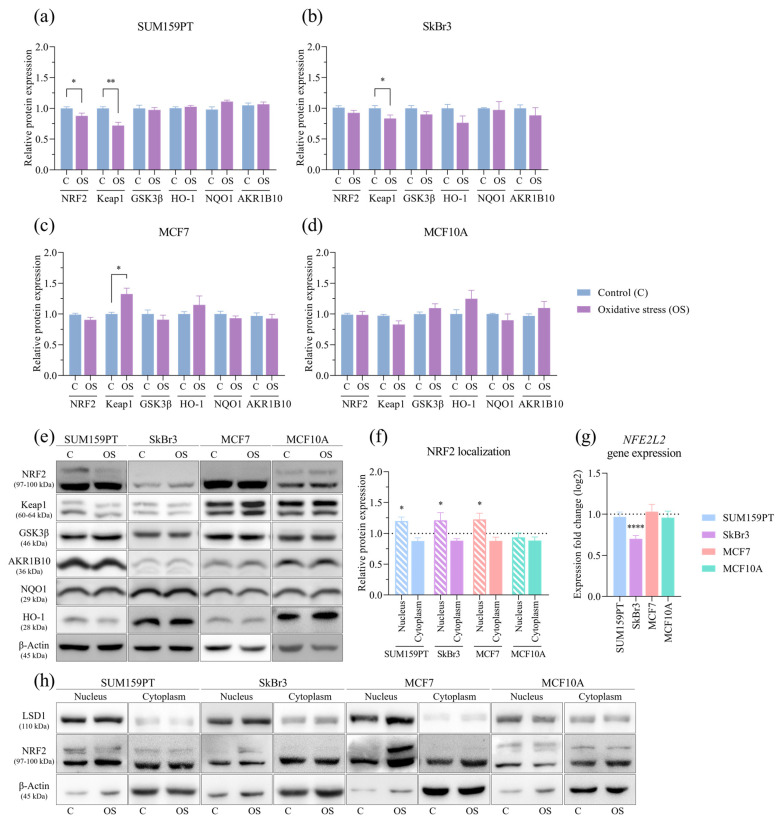
Effect of prolonged exposure to H_2_O_2_ on protein and gene expression. Cells were treated with 20 µM H_2_O_2_ for 14 days, after which proteins were harvested and assayed by Western blotting, and total RNA was isolated, transcribed into cDNA, and analyzed by RT-qPCR. NRF2, Keap1, GSK3β, HO-1, NQO1, and AKR1B10 protein expressions were analyzed in (**a**) SUM159PT, (**b**) SkBr3, (**c**) MCF7, and (**d**) MCF10A. After the same treatment, cytoplasmatic and nuclear protein fractions were isolated and assayed by Western blotting, and (**f**) NRF2 protein localization was analyzed. Protein level is shown as a relative value compared to untreated control. Representative immunoreactive bands are shown in panels (**e**,**h**). *NFE2L2* gene expression is shown in panel (**g**). Expression fold change in a target gene is shown compared to untreated control. Experiments were performed biologically and technically in triplicate. Results are presented as mean ± SEM. * *p* ≤ 0.05, ** *p* ≤ 0.01, and **** *p* ≤ 0.0001 compared to untreated control.

The effect of prolonged exposure to oxidative stress on the whole cell protein expression and cytoplasmatic and nuclear fractions was evaluated by Western blotting. NRF2 expression was reduced only in the SUM159PT cell line (*p* = 0.0489) (Figure 4a), while it did not change in the other cell lines. The expression of the regulator of NRF2 activity, Keap1, was affected by prolonged exposure to oxidative stress only in the cancer cell lines, it was decreased in SUM159PT (*p* = 0.0027) (Figure 4a) and SkBr3 (*p* = 0.0436) (Figure 4b), and it increased in MCF7 (*p* = 0.0359) (Figure 4c) compared to the controls. The expression of another regulator of NRF2 activity, GSK3β as well as the NRF2 downstream targets HO-1, NQO1, and AKR1B10 did not change. Due to the rapid turnover of NRF2, its whole-cell protein expression may not provide accurate insight into its activity; thus, we analyzed its presence in cytoplasmic and nuclear fractions. Compared to the untreated conditions, NRF2 was significantly increased in the nucleus of cells exposed to oxidative stress in the SUM159PT (*p* = 0.0233), SkBr3 (*p* = 0.0357), and MCF7 (*p* = 0.0261) cancer cell lines, while no change was observed in the MCF10A nontumorigenic cell line (Figure 4f). The effect of prolonged exposure to oxidative stress on gene expression was analyzed by RT-qPCR. *NFE2L2* gene expression changed only in the SkBr3 cell line, in which it decreased (*p* ≤ 0.0001) (Figure 4g).

**Figure 5:**
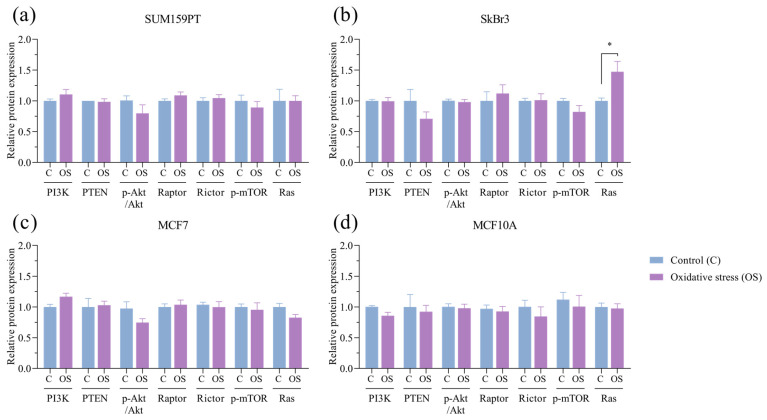
Effect of prolonged exposure to H_2_O_2_ on PI3K, PTEN, pAkt, Akt, Raptor, Rictor, p-mTOR, and Ras protein expression. (**a**) SUM159PT, (**b**) SkBr3, (**c**) MCF7, and (**d**) MCF10A cell lines were treated with 20 µM H_2_O_2_ for 14 days, after which proteins were harvested and assayed by Western blotting. Protein level is shown as a relative value compared to untreated control, and pAkt/Akt is shown as a ratio. Experiments were performed biologically and technically in triplicate. Results are presented as mean ± SEM. * *p* ≤ 0.05 compared to untreated control.

Next, we analyzed the expression profiles of the components within the PI3K/Akt/mTOR signaling pathway. Exposure to oxidative stress did not affect the expression of protein members of the PI3K/Akt/mTOR signaling pathway or PTEN, a PI3K-negative regulator. Akt activity, expressed as the ratio of the protein expression of phosphorylated to unphosphorylated Akt, was also unaffected. We then assessed whether mTOR protein complexes were affected by prolonged exposure to oxidative stress. The protein expression of Raptor, a subunit of mTORC1, and Rictor, a subunit of mTORC2, as well as phosphorylated mTOR, its activated form, did not change due to prolonged exposure to oxidative stress. The only change observed in the signaling pathway protein screening was an increase in Ras expression in the SkBr3 cell line (*p* = 0.0431) (Figure 5b).

**Figure 6:**
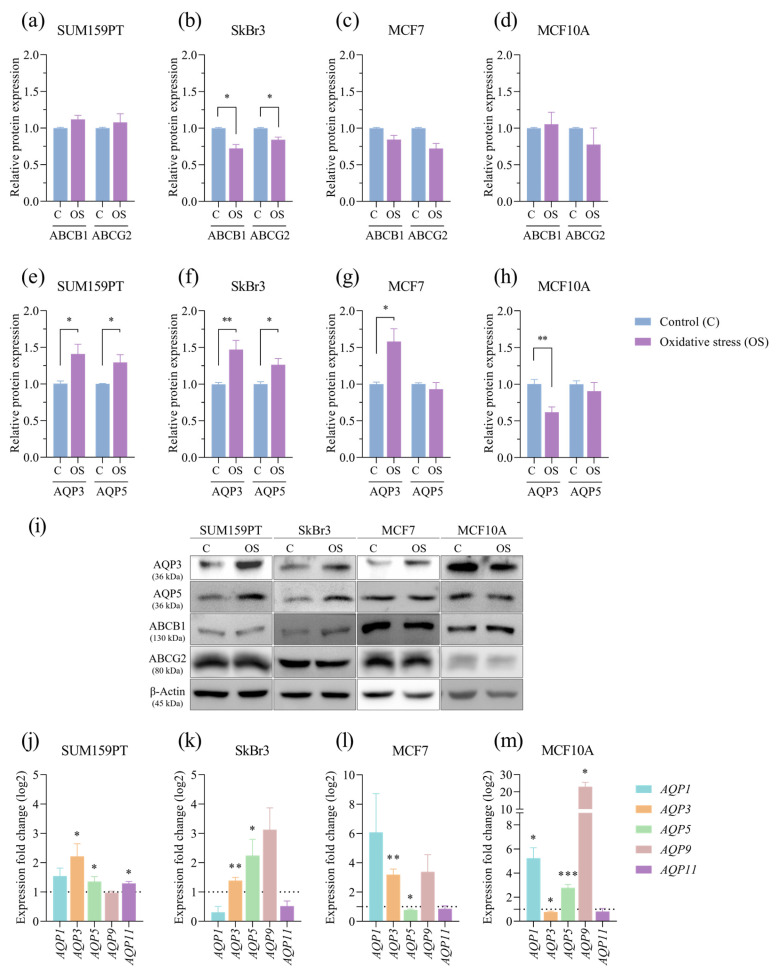
Effect of prolonged exposure to H_2_O_2_ on protein and gene expression. Cells were treated with 20 µM H_2_O_2_ for 14 days, after which proteins were harvested and assayed by Western blotting, and total RNA was isolated, transcribed into cDNA, and analyzed by RT-qPCR. ABCB1 and ABCG2 protein expressions were analyzed in (**a**) SUM159PT, (**b**) SkBr3, (**c**) MCF7, and (**d**) MCF10A, and AQP3 and AQP5 protein expressions in (**e**) SUM159PT, (**f**) SkBr3, (**g**) MCF7, and (**h**) MCF10A. Protein level is shown as a relative value compared to untreated control. Representative immunoreactive bands are shown in panel (**i**). *AQP1*, *AQP3*, *AQP5*, *AQP9*, and *AQP11* gene expressions were analyzed in (**j**) SUM159PT, (**k**) SkBr3, (**l**) MCF7, and (**m**) MCF10A. Expression fold change in a target gene is shown compared to untreated control. Experiments were performed biologically and technically in triplicate. Results are presented as mean ± SEM. * *p* ≤ 0.05, ** *p* ≤ 0.01, and *** *p* ≤ 0.001 compared to untreated control.

To investigate the potential impact of prolonged exposure to oxidative stress on therapy resistance, we analyzed the expression of ABCB1 and ABCG2, ATP-binding cassette (ABC) transporters associated with multidrug resistance. The expression of ABCB1 (*p* = 0.0281) and ABCG2 (*p* = 0.0450) decreased in response to prolonged exposure to oxidative stress in the SkBr3 cell line (Figure 6b), while there were no changes in the others. Next, we tested if prolonged exposure to oxidative stress affected the expression of AQP3 and AQP5 in any of the tested cell lines. In the cancer cell lines, there was an increase in the following expressions: AQP3 (*p* = 0.0330) and AQP5 (*p* = 0.0370) in SUM159PT (Figure 6e), AQP3 (*p* = 0.0053) and AQP5 (*p* = 0.0343) in SkBr3 (Figure 6f), and AQP3 (*p* = 0.0130) in MCF7 (Figure 6g), while in the MCF10A nontumorigenic cell line, the expression of AQP3 (*p* = 0.0015) decreased, and the expression of AQP5 did not change (Figure 6h). Next, we analyzed the expression of selected aquaporin genes to assess changes from prolonged oxidative stress conditions. In addition to proteins, prolonged exposure to oxidative stress also affected *AQP3* and *AQP5* gene expression in all of the tested cell lines. In the cancer cell lines, there was an increase in the expression of *AQP3* (*p* = 0.03) and *AQP5* (*p* = 0.0486) in SUM159PT (Figure 6j), *AQP3* (*p* = 0.0061) and *AQP5* (*p* = 0.0441) in SkBr3 (Figure 6k), and *AQP3* (*p* = 0.0025) in MCF7 and a decrease in *AQP5* expression in MCF7 (*p* = 0.0114) (Figure 6l). In the MCF10A non-tumorigenic cell line, the expression of *AQP3* decreased (*p* = 0.0131), and the expression of *AQP5* increased (*p* ≤ 0.001) (Figure 6m). Besides *AQP3* and *AQP5*, we analyzed the gene expression of other peroxiporins: *AQP1*, *AQP9,* and *AQP11*. In the SUM159 cell line, we observed a significant increase in *AQP11* expression (*p* = 0.041) (Figure 6j), and in the MCF10A cell line, there was an increase in *AQP1* (*p* = 0.0315) and *AQP9* expression (*p* = 0.0127) (Figure 6m).

## 4. Discussion

Our study aimed to unravel the role of aquaporins in the cellular response to oxidative stress. It is known that chronic inflammation and oxidative stress promote all stages of carcinogenesis, and by fine-tuning these processes, cancer cells ensure their survival [34]. Higher expressions of AQP1, AQP3, and AQP5 have been observed in breast cancer, and potential prognostic and predictive characteristics have been suggested [15,16,17,18,19,35]. AQP3 and AQP5 expression correlated with tumor size, lymph node status, and local relapse/distant metastasis in triple-negative breast cancer patients, and patients with higher expressions of these proteins were shown to have worse 5-year disease-free survival and overall survival rates [18]. Premenopausal women have been shown to have higher AQP3 expression compared to postmenopausal women because estrogen directly regulates AQP3 expression by activating the ERE region in the AQP3 gene promoter [36]. Since aquaporins transport H_2_O_2_ and are frequently upregulated in cancer, they have a potential role in maintaining the redox balance within cells and thus impact cancer progression and response to therapy. We used hormone-positive MCF7, HER2-positive SkBr3, and triple-negative SUM159PT breast cancer cell lines, along with the non-tumorigenic MCF10A cell line, to investigate the adaptive responses to prolonged oxidative stress induced by 10 or 20 µM H_2_O_2_. Prolonged exposure to H_2_O_2_ induced cancer-specific responses, resulting in a slight increase in cell viability and/or proliferation in the cancer cell lines. In contrast, the MCF10A non-tumorigenic cell line exhibited a decrease in viability and an increase in proliferation and was more sensitive to 20 µM H_2_O_2_. Since there were no significant differences between the treatments with 10 and 20 µM H_2_O_2_, we chose the latter for further experiments. To assess whether these differences were caused by differences in fatty acid content and the oxidative damage of lipids, we measured lipid hydroperoxides, products of lipid peroxidation, and also fatty acids, substrates for peroxidation. We did not observe a change in either of them. Next, we focused on cell migration. Accelerated cell migration is an important characteristic of cancer, particularly important in cancer invasion and metastasis. Aquaporins have been shown to participate in cell migration and invasion [14] by the reorganization of the actin-cytoskeleton initiated by estrogen-activated AQP3 [36]. Moreover, AQP3 supported cell migration via the H_2_O_2_-dependent activation of the Akt signaling pathway, highlighting the role of AQP3 in H_2_O_2_ transport by colocalization with NADPH oxidase 2, a membrane-based H_2_O_2_ producer, in breast [37], squamous, and lung cancer cell lines [38]. We evaluated cell migration by a wound-healing assay, with the addition of mitomycin C to remove the effect of cell proliferation [39]. ROS stimulates cell migration [40], and we observed this effect in the SkBr3 and MCF7 cell lines, but only after the acute H_2_O_2_ treatment. Prolonged exposure did not affect cell migration in either cell line. Further, knowing that cancer cells have an upregulated antioxidant system to compensate for increased ROS production [41], we analyzed the expression and activity of the major transcription factor responsible for regulating the expression of numerous cytoprotective genes, NRF2. NRF2 protects cells from oxidative damage, a mechanism frequently used by cancer, as the overexpression and/or hyperactivation of NRF2 are often present in various cancers [42]. NRF2 is described as the first responder to ROS [43]; therefore, we analyzed NRF2 levels in whole-cell proteins. NRF2 expression remained unchanged in the whole-cell protein lysates in the SkBr3, MCF7, or MCF10A cell lines and even decreased in the SUM159PT cell line. Confirming these results, our analyses of *NFE2L2* gene expression showed a small, but significant, decrease only in the SkBr3 cell line. Due to its complex regulation and very short half-time, we then turned to its regulator proteins, Keap1 and GSK3β [44]. Interestingly, GSK3β expression did not change due to prolonged exposure to oxidative stress in either of the cell lines tested, while Keap1 expression decreased in the SUM159PT and SkBr3 cell lines and increased in the MCF7 cell line, suggesting a change in this signaling pathway. Hence, we analyzed NRF2 localization in subcellular fractions. We found an NRF2 increase in the nuclear fraction of all three cancer cells exposed to prolonged oxidative stress. Strikingly, there was no change in the non-tumorigenic cell line supporting the Keap1 and GSK3β results as well as the *NFE2L2* gene analysis of this cell line. As NRF2 was increased in the nuclear fraction, suggesting its possible activation, we analyzed the expression of its downstream targets, representing distinct modes of transcription regulation: HO-1 (heme oxygenase 1), NQO1 (NAD(P)H dehydrogenase [quinone] 1), and AKR1B10 (aldo-keto reductase family 1-member B10). Even though NRF2 was increased in the nuclei of the cancer cells after prolonged exposure to oxidative stress, it had no effect on the target proteins. Therefore, our focus turned to non-conventional NRF2 targets: ABC transporters [45]. ABC transporters are membrane proteins involved in multidrug resistance [46]. We analyzed the expression of ABCB1, also known as P-glycoprotein, and ABCG2, i.e., a breast-cancer-resistant protein. Although some studies link NRF2 and MDR proteins [45,47,48,49], the only observed change was a decrease in the expression of both proteins in the SkBr3 cell line.

Finally, we analyzed the expression of aquaporins involved in the transport of H_2_O_2_, AQP3, and AQP5. After prolonged exposure to oxidative stress in all cell lines, the aquaporin expression changed at both the protein and transcriptional levels. AQP3 showed the same pattern in the cancer cell lines; specifically, it increased in the cancer cell lines and decreased in the MCF10A non-tumorigenic cell line. AQP5 expression distinctively varied between the cancer cell lines. It increased in the SUM159PT, SkBr3, and MCF10A cell lines, while in the ER-positive MCF7cell line, it slightly decreased. To investigate which signaling pathway was involved in the regulation of aquaporin expression, we analyzed the PI3K/Akt/mTOR signaling pathway, which has already been shown to be involved [37,50], but we did not observe treatment-induced changes in any of the analyzed protein members: PI3K, PTEN, phosphorylated or unphosphorylated Akt, Raptor, Rictor, or phosphorylated mTOR. The only change observed was an increase in Ras expression in the SkBr3 cell line, which is in accordance with the literature data in which AQP5 triggers Ras signaling in NIH3T3 cells and also in breast cancer [51,52]. Additional research is needed to elucidate the signaling pathways regulating aquaporin expression. In addition to AQP3 and AQP5, which are the most frequently mentioned overexpressed aquaporins/peroxiporins in the context of breast cancer, we analyzed the gene expression of other peroxiporins: *AQP1*, *AQP8*, *AQP9*, and *AQP11*. *AQP1* was differently expressed in different cell lines, with a statistically significant increase observed only in the MCF10A cell line, along with an increase in *AQP9* expression. In our previous paper, we reported a higher expression of *AQP11* in cancer cell lines [50], while in conditions of prolonged oxidative stress, this expression increased only in the SUM159PT cell line. In our cell lines, we did not detect transcripts of *AQP8*, the last of the peroxiporin group. Aquaporin expression changed in a cell-type-specific manner and is affected by H_2_O_2_. While some aquaporins increase, others decrease within the same cell line, highlighting the importance of investigating their regulation as well as interdependence. We showed that the expression of AQP3 and/or AQP5 was increased in all cancer cell lines, thereby emphasizing their role in breast cancer. In support of our results is a study showing that silencing AQP3 expression in the MDA-MB-231 breast cancer cell line results in a decrease in cell proliferation, migration, and invasion and an increase in cell death by 5-fluorouracil [53]. AQP5 expression was associated with enhanced proliferation and migratory potential in lung cancer cell lines via the EGFR/ERK/p38 MAPK pathway [54], while silencing or exposure to hyperosmotic stress reduced AQP5 expression, leading to decreased MCF7 cell proliferation and migration [55]. Aquaporins have the potential to serve as a prognostic factor and a therapeutic target due to their role in cell proliferation, migration, and therapy response. For example, several studies have pointed to aquaporins’ role in modulating cellular responses to different chemotherapeutics. The overexpression of AQP1, AQP3, or AQP5 in breast cancer cell spheroids caused a reduction in cell viability in response to cisplatin, 5-fluorouracil, or doxorubicin [56]. Yet, they are associated not only with therapy resistance but also with sensitivity. AQP1 overexpression is associated with increased sensitivity to anthracycline therapy in invasive ductal carcinoma patients [57], whereas silencing AQP3 disrupts the drug-induced cell cycle arrest [58]. In addition, AQP3 expression was upregulated in response to nucleoside-derived drugs in breast and colon cancer cell lines, pointing to an adaptive cellular response to the treatment [58]. AQP5 was also shown to be involved in therapy response because AQP5 silencing in the doxorubicin-resistant MCF7 breast cancer cell line, characterized by upregulated AQP5 expression, reversed drug resistance, inhibited proliferation, and induced apoptosis [59]. Silencing AQP5 in colorectal cancer confirmed an AQP5-dependent drug response by increasing the level of chemosensitivity to 5-fluorouracil through the suppression of the Wnt-β-catenin signaling pathway [60]. Additionally, AQP5 silencing led to a reduction in the expression levels of drug-resistance-related proteins, including P-glycoprotein (P-gp) [59]. Here, we did not observe changes in MDR-related pumps as a response to prolonged oxidative stress, although AQP5 was increased. Our results highlight the modulation of aquaporins by H_2_O_2_ as a cellular response to oxidative stress. Interestingly, the observed changes are associated with cancer cell lines, without alternations in the non-tumorigenic cell line. Yet, the association of these changes with a specific signaling pathway is still a challenge. Furthermore, the differences observed between the cancer cell lines representing different breast cancer subtypes point to differences in the biology of these subtypes. Therefore, this research should be extended to study specific subtypes of breast cancer using multiple cell lines, rather than relying on a single cell line per subtype. Our results together with the literature data suggest the potential regulation of aquaporins by chemotherapeutics mediated, at least in part, by oxidative stress. Therefore, understanding the pathways activated by oxidative stress and the modulation of aquaporins could elucidate the mechanisms underlying therapy resistance. Understanding these mechanisms could enable the use of aquaporins in clinical practice: they could serve either as an indicator of therapy effectiveness or could be a new target of counteracting therapy resistance.

## 5. Conclusions

To conclude, our study implies the modulation of aquaporins by H_2_O_2_ as a cellular response to prolonged oxidative stress in cancer cell lines. Still, further research is needed to elucidate the mechanisms of aquaporin modulation in cancer biology and therapy resistance.

## Figures and Tables

**Table 1:** Primer sequences used for quantitative reverse transcription PCR (RT-qPCR) analysis.

*AQP1* (NM_198098)	Forward	AATGACCTGGCTGATGGTGT
	Reverse	CAGGAGGTGTCCAAGGGCTA
*AQP9* (NM_020980)	Forward	TCTCAGTCGAGGACGTTTTGG
	Reverse	GTGACCACCAGAGACACCG
*AQP11* (NM_173039)	Forward	TGCAGGAGGAAGTCTAACAGG
	Reverse	AGCCATGGAAGGAAAAAGCTG
*B2M* (NM_004048)	Forward	TGTCTTTCAGCAAGGACTGGT
	Reverse	ACATGTCTCGATCCCACTTAAC
*PPIA* (NM_001300981)	Forward	GACTGAGTGGTTGGATGGCA
	Reverse	GCTCCATGGCCTCCACAATA
*HPRT-1* (NM_000194)	Forward	CCCTGGCGTCGTGATTAGTG
	Reverse	TCGAGCAAGACGTTCAGTCC

## Data Availability

The data is contained within this article.
